# Loss of histone H4K20 trimethylation predicts poor prognosis in breast cancer and is associated with invasive activity

**DOI:** 10.1186/bcr3681

**Published:** 2014-06-22

**Authors:** Yuhki Yokoyama, Ayaka Matsumoto, Miki Hieda, Yoshimi Shinchi, Eri Ogihara, Mai Hamada, Yu Nishioka, Hiroshi Kimura, Katsuhide Yoshidome, Masahiko Tsujimoto, Nariaki Matsuura

**Affiliations:** 1 Osaka University Graduate School of Medicine and Health Science, 1-7 Yamadaoka, Suita City, Osaka 565-0871, Japan; 2 Osaka University Graduate School of Frontier Science, 1-3 Yamadaoka, Suita City, Osaka 565-0871, Japan; 3 Department of Breast Surgery, Osaka Police Hospital, 10-31, Kitayamacho, Osaka City, Osaka 543-0035, Japan; 4 Department of Pathology, Osaka Police Hospital, 10-31, Kitayamacho, Osaka City, Osaka 545-0035, Japan

## Abstract

**Introduction:**

Loss of histone H4 lysine 20 trimethylation (H4K20me3) is associated with multiple cancers, but its role in breast tumors is unclear. In addition, the pathological effects of global reduction in H4K20me3 remain mostly unknown. Therefore, a major goal of this study was to elucidate the global H4K20me3 level in breast cancer tissue and investigate its pathological functions.

**Methods:**

Levels of H4K20me3 and an associated histone modification, H3 lysine 9 trimethylation (H3K9me3), were evaluated by immunohistochemistry in a series of breast cancer tissues. Univariate and multivariate clinicopathological and survival analyses were performed. We also examined the effect of overexpression or knockdown of the histone H4K20 methyltransferases, SUV420H1 and SUV420H2, on cancer-cell invasion activity *in vitro*.

**Results:**

H4K20me3, but not H3K9me3, was clearly reduced in breast cancer tissue. A reduced level of H4K20me3 was correlated with several aspects of clinicopathological status, including luminal subtypes, but not with HER2 expression. Multivariate analysis showed that reduced levels of H4K20me3 independently associated with lower disease-free survival. Moreover, ectopic expression of SUV420H1 and SUV420H2 in breast cancer cells suppressed cell invasiveness, whereas knockdown of SUV420H2 activated normal mammary epithelial-cell invasion *in vitro*.

**Conclusions:**

H4K20me3 was reduced in cancerous regions of breast-tumor tissue, as in other types of tumor. Reduced H4K20me3 level can be used as an independent marker of poor prognosis in breast cancer patients. Most importantly, this study suggests that a reduced level of H4K20me3 increases the invasiveness of breast cancer cells in a HER2-independent manner.

## Introduction

The heterogeneous nature of breast cancer has been well established [1]. Various types of prognostic factors such as nuclear grade, hormone receptor status, human epidermal growth factor receptor (HER2) expression, and MIB-1 index have been used to determine therapeutic approaches. In addition, breast tumors can be classified into subtypes based on their expression profile, and each subtype is associated with distinct histological markers and clinical parameters: luminal A (estrogen receptor alpha (ER)- and/or progesterone receptor (PgR)-positive, HER2-negative), luminal B (ER- and/or PgR-positive, HER2-positive), HER2 (ER- and PgR-negative, HER2-positive), basal-like (ER-, PgR-, and HER2-negative; cytokeratin 5- and cytokeratin 6 (CK5/6)-positive and/or epidermal growth factor receptor (EGFR)-positive), and unclassified (negative for all five markers) [2,3]. Luminal types (luminal A and B) are associated with less aggressive metastatic disease and longer disease-free survival [4]; within luminal types, Luminal B is associated with poorer survival [5]. However, more effective markers for prediction of patients’ outcomes are still needed.

Epigenetic alterations such as DNA methylation and histone modifications occur in many cancers (reviewed in [6-9]). Aberrant histone modifications are associated with carcinogenesis and cancer progression, and global histone modification patterns can predict clinical outcome, as recently shown for many types of cancer [10-12]. Loss of histone H4 lysine 20 trimethylation (K20me3) is considered a hallmark of human cancer and a potential prognostic marker in many types of cancer other than breast cancer [10,13-15].

The loss of H4K20me3 has been observed in animal models of breast carcinogenesis [16] and H4K20me3 levels are reduced in malignant breast cancer-derived cell lines relative to those in nontumorigenic breast epithelial cells [17]. The data, however, are not entirely consistent: at least one study reported that H4K20me3 was present at relatively high levels in the majority of breast-tumor cases [18]. Therefore, the H4K20me3 level in breast cancer is not well defined, and the clinical significance of H4K20me3 as an independent factor in breast cancer is not understood. This study aimed to determine whether the level of H4K20me3 is reduced in breast cancer, whether H4K20me3 level is associated with clinicopathological data, and whether H4K20me3 is an independent prognostic marker. We observed that H4K20me3 level was reduced in cancer cells in breast-tumor tissue, and that loss of H4K20me3 significantly correlated with some aspects of clinicopathological status; in particular, loss of H4K20m3 predicted poor prognosis in patients. Furthermore, we also showed that H4K20me3 is associated with behavior of breast cancer cell, specifically in regard to invasive potential.

## Materials and methods

### Immunohistochemical staining

Samples were obtained from benign and tumor tissue from 112 patients with breast cancer who underwent operations between 2000 and 2004 at Osaka Police Hospital (Osaka, Japan). The samples were immunostained with the informed consent of patients and the approval of the Osaka Police Hospital Ethics Committee according to the institutional ethics and legal rules. The detailed clinical subtypes of these patients are enumerated in Table S1 in Additional file 1. Formalin-fixed, paraffin-embedded specimens were stained as described previously [19]. Sections (2 μm thick) were deparaffinized in xylene, dehydrated in a graded series of ethanol, and processed for antigen retrieval in 0.01 M citrate buffer using a Pascal pressure chamber (DAKO, Glostrup, Denmark). Endogenous peroxidase was blocked in 3% H_2_O_2_ in methanol. The sections were blocked with 5% bovine serum albumin (BSA) and incubated with primary monoclonal antibodies (mAbs) against H3K9me3 (CMA318) and H4K20me3 (CMA423); the performance of these antibodies has been validated in Western blotting, immunofluorescence microscopy, and enzyme-linked immunosorbent assay (ELISA) ( [20,21], and Hayashi-Takanaka *et al*., (manuscript to be submitted). Next, sections were incubated with a biotinylated anti-mouse immunoglobulin G (IgG) antibody (DAKO) and further incubated with peroxidase-conjugated streptavidin (DAKO). Samples were visualized with 3,3-diaminobenzidine (DAB) solution (Sigma-Aldrich, St Louis, MO, USA) and counterstained with hematoxylin. The immunohistochemical staining of tissues were assessed by at least two pathologists.

### Staining scores

**Figure 1:**
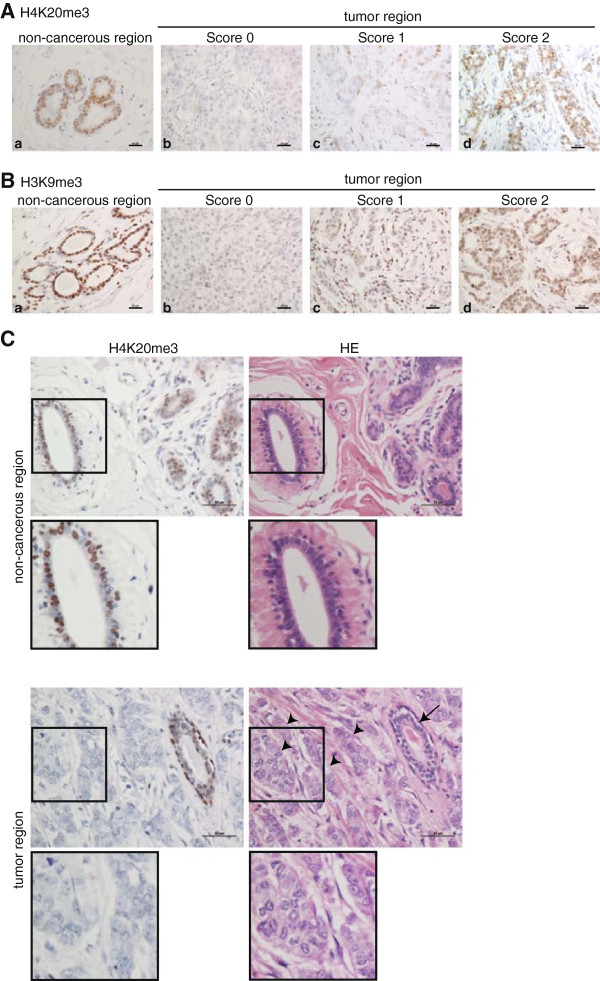
**Immunohistochemical staining for H4K20me3 and H3K9me3. (A and B)** Specimens of breast tumor were stained using anti-H4K20me3 **(A)** and anti-H3K9me3 **(B)** mAbs. Representative cases of noncancerous (a) and cancerous regions (b, c, d) are shown. Staining of cancerous regions was assessed using a three-point scoring system based on the percentage of positively stained cells. Bar, 20 μm. **(C)** H4K20me3 staining of tumor and nontumorigenic region. Within each specimen, the tumor region included cancerous and noncancerous regions. A high level of H4K20me3 staining in noncancerous regions (arrow) in comparison with the surrounding malignant cells (arrowhead) was observed. The lower panels were acquired at higher magnification. Bar, 50 μm. H & E, hematoxylin and eosin staining; mAbs, monoclonal antibodies.

Immunohistochemical staining of tissues was evaluated based on the percentage of cancer cells stained. Either intermediate or strong nuclear staining was considered positive. The percentage of positive cancer cells was graded using the following categories: negative (score 0) = less than 5% of cancer cells positively stained; weak positive (score 1) = 5 to 20% of cancer cells positively stained; strong positive (score 2) = more than 20% of cancer cells positively stained. Noncancerous mammary epithelial cells were scored as 2 (Figure 1A, a), and could be used as internal positive controls for staining.

### Statistical analysis

Results were tabulated as a Microsoft Excel worksheet, and then exported into and analyzed in Ekuseru-Toukei 2012 (Social Survey Research Information Co., Ltd., Tokyo, Japan). For clinicopathological analysis (Table 1), the chi-squared test was applied. Univariate survival analyses were carried out according to Kaplan-Meier and log-rank tests. A multivariable Cox proportional hazard regression analysis was performed by using the JMP software version 10 (SAS Institute Inc., Cary, NC, USA). MIB-1 index were excluded because they correlated significantly with H4K20me3 level (Table 1, *P* <0.01). Multicollinearity within these predictor variables was examined by nonparametric test (Spearman’s rank correlation coefficient) using all combination (JMP software). PgR status and intrinsic subtype, were excluded because they correlated significantly with ER (Spearman’s rank correlation coefficient was 0.709 and 0.904, respectively). Lymph node metastasis was also excluded because the number of samples exhibiting such metastasis was too small to analyze. Therefore, H4K20me3, nuclear grade, ER, and HER2 status were used for the multivariate analysis described in Table 2. Results were considered statistically significant when the *P* value from a two-tailed test was <0.05.

**Table 1:** Clinicopathological parameters in patients with breast cancer

		**H3K9me3**						**H4K20me3**			
	**score**	**0**	**1+**	**2+**			**score**	**0**	**1+**	**2+**	
	**N = 71**	**25**	**22**	**24**			**N = 93**	**36**	**23**	**34**	
		**35.2**	**31.0**	**33.8**	**(%)**			**38.7**	**24.7**	**36.6**	**(%)**
	**N**				***P*** *		**N**				***P*** *
**Pathological classification**	71				0.503	**Pathological classification**	93				0.478
DCIS, microinvasive		1	5	3		DCIS, microinvasive		1	3	4	
idc, pap		9	9	7		idc, pap		11	8	10	
idc, sol		4	3	3		idc, sol		10	3	3	
idc, sci		8	2	6		idc, sci		8	4	10	
special type		3	3	5		special type		6	5	7	
**Lymph node metastasis**	64				0.493	**Lymph node metastasis**	83				0.060
0-3		20	15	16		0-3		22	19	25	
3<		3	4	6		3<		11	3	3	
**Nuclear grade**	70				0.838	**Nuclear grade**	91				**0.022**
1		12	12	9		1		8	10	21	
2		4	4	4		2		7	4	6	
3		9	6	10		3		19	9	7	
**ER**	70				0.707	**ER**	93				**0.027**
Negative		9	9	11		Negative		21	10	9	
Positive		16	13	12		Positive		15	13	25	
**PgR**	68					0.590	**PgR**	91				**0.019**
Negative		10	9	12		Negative		21	13	10		
Positive		14	13	10		Positive		13	10	24		
**HER2**	71				0.580	**HER2**	93				0.082	
Negative (0/1)		20	15	19		Negative (0/1)		29	14	29		
Positive (2/3)		5	7	5		Positive (2/3)		7	9	5		
**Intrinsic subtype**	69				0.462	**Intrinsic subtype**	91				**0.002**	
Luminal		18	14	12		Luminal		14	14	28		
Non-luminal		7	8	10		Non-luminal		20	9	6		
**MIB-1 index**	69				0.482	**MIB-1 index**	91				**0.001**	
<30		14	9	9		<30		9	8	24		
30≦		11	13	13		30≦		25	15	10		

*Chi-squared test. Bold indicates values that are statistically significant. DCIS, ductal carcinoma *in situ*; idc, invasive ductal carcinoma; pap, papillotubular carcinoma; sol, solid-tubular carcinoma; sci, scirrhous carcinoma; ER, estrogen receptor; PgR, progesterone receptor; luminal, ER positive and/or PgR positive.

**Table 2:** Multivariate analysis of clinicopathological parameters

	**Overall survival**			**Disease-free survival**		
**Variables**	**HR**	**(95% Cl)**	***P*** *	**HR**	**(95% Cl)**	***P*** *
H4K20me3 score (score2 : score 0,1)	2.85	(0.52 to 53.37)	0.263	3.37	(1.09 to 14.80)	0.033
Nuclear grade (G1, G2 : G3)	5.60	(1.19 to 41.07)	0.028	2.52	(0.97 to 6.75)	0.059
ER (positive : negative)	0.93	(0.18 to 3.70)	0.920	1.70	(0.65 to 4.38)	0.272
HER2 (positive : negative)	2.62	(0.67 to 9.30)	0.158	1.43	(0.46 to 3.70)	0.509

*Cox proportional hazards model was used to assess the independent prognostic contribution of H4K20me3 after accounting for other potentially important covariates. HR, hazard ratio; Cl, confidence interval.

### Cell culture, transfection, and siRNA knockdown

The human breast cancer cell lines HBL-100, MDA-MB-231, and BT-474 (American Type Culture Collection) were grown in Dulbecco’s modified Eagle’s medium (DMEM) (Nissui Seiyaku, Tokyo, Japan) supplemented with 10% (w/v) fetal bovine serum (FBS) (Biowest, Kansas City, MO, USA), 100 U/ml penicillin, and 100 μg/ml streptomycin (Life Technologies, Carlsbad, CA, USA). MCF-7 cells were grown in RPMI1640 (Nissui Seiyaku) supplemented with 10% (w/v) FBS, 100 U/ml penicillin, and 100 μg/ml streptomycin. MCF10A mammary epithelial cells were grown in DMEM/F12 (Life Technologies) supplemented with 5% (w/v) horse serum (Life Technologies), 20 ng/ml epidermal growth factor (EGF) (PeproTech, Rocky Hill, NJ, USA), 0.5 μg/ml hydrocortisone (Sigma-Aldrich), 100 ng/ml cholera toxin (Bio Academia, Osaka, Japan), and 10 μg/ml insulin (Wako, Osaka, Japan). Cells were purchased from ATCC via Sumitomo Pharmaceuticals International (Osaka, Japan); stocks were made at passage 2 or 3, and cells were used for fewer than 15 passages. Cells were transfected using Lipofectamine LTX (Life Technologies), Lipofectamine 2000 (Life Technologies), or Polyethylenimine ‘Max’ (Polysciences, Inc., Warrington, PA, USA). Small interfering RNAs (siRNAs) against the *SUV420H2* coding region (siGENOME SMARTpool siRNAs, a mixture of #1, GUGAAGGUGCUCCGGGACA; #2, GCGGUGAAGAGCUGUGACA; #3, CGACAGAGUGACAGCACGA; and #4, CUCAGCGCUGGAAACUUU) and negative-control siRNAs (siGENOME nontargeting siRNA pool, a mixture of four nontargeting siRNAs) were obtained from Thermo Fisher (Waltham, MA USA) and transfected into cells using RNAiMax (Life Technologies).

### Immunofluorescence microscopy and intensity measurement

Cells grown on glass coverslips were fixed and permeabilized for 10 minutes with 4% paraformaldehyde containing 0.5% Triton X-100, and then blocked with 5% BSA. Next, cells were incubated with a primary antibody against H4K20me3 and appropriate secondary antibodies (Jackson ImmunoResearch Laboratories, West Grove, PA, UK). Finally, coverslips were mounted in ProLong Gold Antifade Reagent with 4′,6-diamidino-2-phenylindole (DAPI) (Life Technologies). Fluorescence intensity was analyzed using a fluorescence microscope (IX81; Olympus, Tokyo, Japan). The staining intensities were measured using MetaMorph version 7.1 (Molecular Devices, Sunnyvale, CA, USA).

### Construction of plasmids

Human *SUV420H1* and *SUV420H2* cDNAs were amplified from HBL-100 cDNA and inserted into the *Xho*I/*Bam*HI sites and *Eco*RI/*Bam*HI sites of vector pEGFP-C1, respectively. PCR reactions were performed using KOD Plus high-fidelity DNA polymerase (Toyobo, Osaka, Japan). All cDNA constructs were verified by DNA sequencing.

### Real-time PCR

Total RNA was extracted from normal epithelial and breast cancer cell lines as previously described [22]. The mRNA levels of *SUV420H1* and *SUV420H2* were quantitated by real-time PCR using a LightCycler 480 System (Roche Diagnostics, Basel, Switzerland) and normalized to the mRNA level of *GAPDH* (encoding glyceraldehyde-3-phosphate dehydrogenase). Experiments were performed in triplicate. Sequences of the primers used are as follows: for *SUV420H1*, 5′-GCACGGCACTATTTTCTCAA-3′ and 5′-TCCACTGTCAGTTGCAAACA-3′; for *SUV420H2*, 5′-GGCCCGCTACTTCCAGAG-3′ and 5′-GCAGGATGGTAAAGCCACTT-3′; for *GAPDH*, 5′-CAATGACCCCTTCATTGACC-3′ and 5′-TTGATTTTGGAGGGATCTCG-3′.

### Invasion assay

Matrigel invasion assay was performed using Chemotaxicells (8-μm pore size; Kurabo, Osaka, Japan) as described previously [23]. Briefly, transwell inserts were coated with 100 μg/ml matrigel (BD Biosciences, Franklin Lakes, NJ, USA), which contains laminin, type IV collagen, and perlecan; 10% FBS was used as chemoattractant. Twenty-four hours after transfection, 2 × 10^5^ or 1 × 10^5^ cells were added to the upper chamber and incubated for 24 or 48 hours (for MDA-MB-231 and BT-474 cells, respectively). Cells were fixed with 3.7% formaldehyde and stained with hematoxylin. Non-invading cells on the upper surface were removed by scrubbing with a cotton swab, and the invading cells were counted under a microscope (200×). Each assay was carried out in triplicate.

For invasion assays using MCF10A, cells were transfected with *SUV420H2*-targeted siRNA or nontargeted negative control siRNA. Twenty-four hours after transfection, cells were seeded in the upper chamber of transwells. After an additional 48 hours (a total of 72 hours after siRNA transfection), cells were fixed, stained, and counted in five randomly chosen microscopic fields per sample.

## Results

### Loss of H4K20me3 in breast cancer tissues

Loss of H4K20me3 has been reported in multiple types of human tumors [10,13-15]. To date, however, no study has demonstrated reduction of H4K20me3 in breast tumors. To address this issue, we performed immunohistochemistry with breast-tumor tissues using a mAb against H4K20me3. Clear and strong H4K20me3 staining was observed in mammary epithelial and myoepithelial cells in the noncancerous acini of terminal-duct lobular units adjacent to cancer cells (Figure 1A, a and C), as well as in benign tumor tissues (Figure S1 in Additional file 2), whereas various staining intensities were observed in cancer cells (Figure 1A, b-d). The staining intensity in the tumor cells in each case was homogenous, but the intensities varied between individual cases. In order to facilitate comparisons among cancer cases, we used a three-point scoring system based on the percentage of positively stained cancer cells (Figure 1A): score 0, less than 5% of cancer cells positively stained; score 1, 5 to 20% of cancer cells positively stained; score 2, more than 20% of cancer cells positively stained. Either intermediate or strong nuclear staining was considered as positive. Score was evaluated based on staining in cancer cells, but not in adjacent noncancerous acini. More than half of cancer cases (59 of 93, 63.4%) scored as 0 or 1 (Table 1, upper right), indicating a reduction of H4K20me3 staining in cancer cells relative to the adjacent noncancerous regions, which stained positive and were scored as 2 (Figure 1A, a and C). A single specimen often exhibited different levels of staining between noncancerous and cancerous regions (Figure 1C, lower).

### Decreased H4K20me3 level is associated with clinicopathological status

Although loss of H4K20me3 was observed in many cancer cases (63.4%), in 36.6% of cases more than 20% of cancer cells stained positively (Table 1, upper right), suggesting that the differences in the H4K20me3 levels among different tumors might be related to clinicopathological factors. In order to examine this possibility, we evaluated the correlation between histone H4K20me3 levels and various clinicopathological data using the semi-proportional score described above. As shown in Table 1, H4K20me3 status was not associated with pathological classification such as ductal carcinoma *in situ* (DCIS), invasive ductal carcinoma (IDC), or more detailed classification of IDC [24], but it was negatively correlated with nuclear grade (*P* <0.05) and MIB-1 index (*P* <0.01). H4K20me3 status also positively correlated with ER expression (*P* <0.05) and PgR expression (*P* <0.05), but not with HER2 expression. H4K20me3 staining score associated with each subtype (*P* <0.01, Table S2 in Additional file 3), although H4K20me3 did not associate with Luminal A/Luminal B distribution (*P* = 0.482, Table S3 in Additional file 4). Therefore, we analyzed the association between H4K20me3, luminal subtype, and non-luminal subtype (Table 1). Luminal intrinsic subtype (ER- and/or PgR-positive) was also significantly correlated with loss of H4K20me3 (*P* <0.01). These results implied that the diversity of H4K20me3 staining patterns might result from the heterogeneous nature of breast cancer, as reflected by these aspects of clinicopathological status.

### H3K9me3 does not correlate with clinicopathological status

H4K20me3 is a heterochromatic mark, as is H3K9me3. Formation of H4K20me3 requires previous formation of H3K9me3 by the SUV39H1 and SUV39H2 enzymes [25,26]. In several cancers, such as gastric adenocarcinoma and non-small cell lung cancer, H3K9me3 staining positively correlates with cancer recurrence and poor survival rate [27,28]; consistent with this, in a mouse model of colorectal cancer, H3K9me3 level is elevated in invasive regions and drives tumorigenesis [19]. Therefore, we also evaluated H3K9me3 staining in breast cancer tissue. Similar to H4K20me3 staining, all mammary epithelial and myoepithelial cells in noncancerous acini were stained by an H3K9me3 mAb (Figure 1B, a), but diverse staining patterns were observed in cancerous regions (Figure 1B, b-d). However we could not find any association between H3K9me3 status and clinicopathological data (Table 1, left). In these experiments, the evaluation of immunohistochemical staining levels was performed as described above for H4K20me3.

### H4K20me3 level is associated with patient disease-free survival

**Figure 2:**
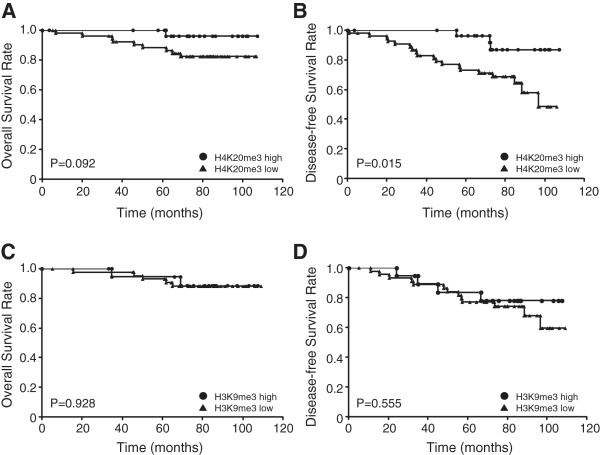
**Kaplan-Meier analysis of H4K20me3 and H3K9me3 levels in breast cancer patients.** The three-point scoring system used in Figure 1 was consolidated into a two-point scale. Cases with scores of 0 and 1 constitute the low-staining group, and cases with a score of 2 constitute the high-staining group. Then patient overall survival time **(A and C)** and disease-free survival rate **(B and D)** were compared between the H4K20me3 low- and high-staining groups **(A and B)**, and between the H3K9me3 low- and high-staining groups **(C and D)**, by Kaplan-Meier analysis. This method yields a survival curve that considers both outcomes (death) and censored cases. Significance is calculated by log-rank test between two groups (low- and high-staining).

Next we compared overall and disease-free survival rates among cases with different levels of H4K20me3 (Figure 2A and B) and H3K9me3 (Figure 2C and D), using the log-rank test. In this analysis, the three-point scoring system described above (Figure 1A) was consolidated into a two-point scale: cases with scores of 0 and 1 constitute the low-staining group, and cases with a score of 2 constitute the high-staining group. For H4K20me3, the high-staining group had higher overall and disease-free survival rates than the low-staining group (Figure 2A). In particular, H4K20me3 staining score positively associated with disease-free survival rate in a statistically significant manner. ER status positively associated with overall and disease-free survival rate, but this relationship was not statistically significant (Figure S2 in Additional file 5). On the other hand, as with the clinicopathological data, the H3K9me3 staining pattern did not show any association with either overall or disease-free survival rate (Figure 2C and D). To further evaluate the potential prognostic value of clinical variables including the H4K20me3 score, we performed a multivariable Cox proportional hazards regression analysis using H4K20me3, nuclear grade, ER, and HER2 expression. As expected, increasing nuclear grade was correlated with overall survival (hazard ratio for recurrence = 5.60, 95% confidence interval = 1.19 to 41.07; *P* <0.05, Table 2). The result also showed that lower H4K20me3 score was associated with a significant decrease in disease-free survival (hazard ratio for recurrence = 3.37, 95% confidence interval = 1.09 to 14.80; *P* <0.05, Table 2). These results demonstrate that H4K20me3 is an independent prognostic marker for disease-free survival in breast cancer.

### Upregulation of H4K20me3 represses breast cancer cell invasion

**Figure 3:**
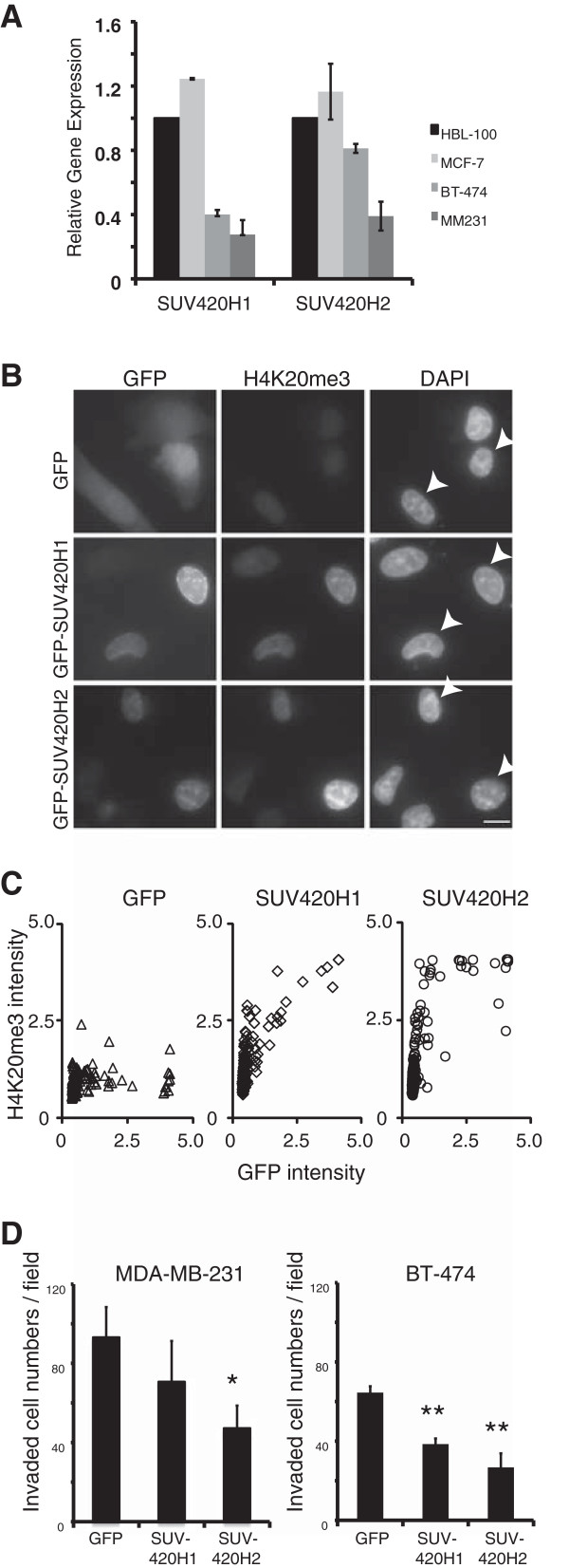
**Overexpression of SUV420H1 and SUV420H2 represses breast cancer cell invasion. (A) ***SUV420H1* and *SUV420H2* mRNA expression levels in a several normal and breast cancer cell lines were examined by real-time PCR. The relative levels of *SUV420H1* and *SUV420H2* mRNA were normalized against the level of *GAPDH* mRNA. Average values with standard deviation are shown. **(B)** To assess the effects of SUV420H1 and SUV420H2 overexpression on the level of H4K20me3, MDA-MB-231 cells were transfected with GFP-tagged SUV420H1 and SUV420H2. pEGFPC1, which carries GFP alone, was used as the empty-vector (negative) control. Cells were fixed, visualized by GFP fluorescence, and simultaneously stained with anti-H4K20me3 mAb. Cells were counterstained with DAPI. Histone H4K20 trimethylation was increased by SUV420H1 and SUV420H2 expression. Arrowheads indicate SUV420H1- or SUV420H2-transfected cells. Bar: 20 μm. **(C)** MDA-MB-231 cells were transfected with the indicated plasmids. The intensity of GFP and H4K20me3 staining were measured (n = 200) using MetaMorph version 7.1. X axis shows GFP intensity. Y axis shows H4K20me3 intensity. **(D)** Overexpression of SUV420H1 and SUV420H2 represses breast cancer cell invasion. GFP, GFP-SUV420H1, or GFP-SUV420H2 were transfected into MDA-MB-231 or BT-474 cells, and invasive capacities were measured using matrigel-coated Chemotaxicells. Results are presented as means ± standard deviation (SD). * and ** indicate significant differences (*P* <0.05 and *P* <0.01, respectively) relative to the mock transfectant.

Both the univariate and multivariate analyses demonstrated that loss of H4K20me3 staining is associated with shorter disease-free survival (Figure 2 and Table 2). Therefore, we focused on the H4K20me3-specific histone methyltransferases SUV420H1 and SUV420H2. According to the public database MENT (Methylation and Expression database of Normal and Tumor tissues; [29,30]), whose contents were obtained from selected datasets from GEO (Gene Expression Omnibus) and TCGA (The Cancer Genome Atlas), breast cancer tissues tend to express lower levels of SUV420H2 than normal breast tissue. Furthermore, real-time PCR analysis revealed that invasive cells, such as MDA-MB-231 or BT-474, tend to express lower levels of SUV420H1 and SUV420H2 than other breast cancer cells (Figure 3A). Immunofluorescence microscopy revealed that ectopic expression of both enzymes could upregulate H4K20me3 (Figure 3B). Nuclei lacking exogenous expression of SUV420H1 or SUV420H2 exhibited very faint H4K20me3 staining (Figure 3B). To quantitate this observation, we plotted H4K20me3 staining intensity against GFP intensity (Figure 3C). The result revealed that H4K20me3 level was positively correlated with the levels of ectopically expressed SUV420H1 and SUV420H2 (Figure 3C).

**Figure 4:**
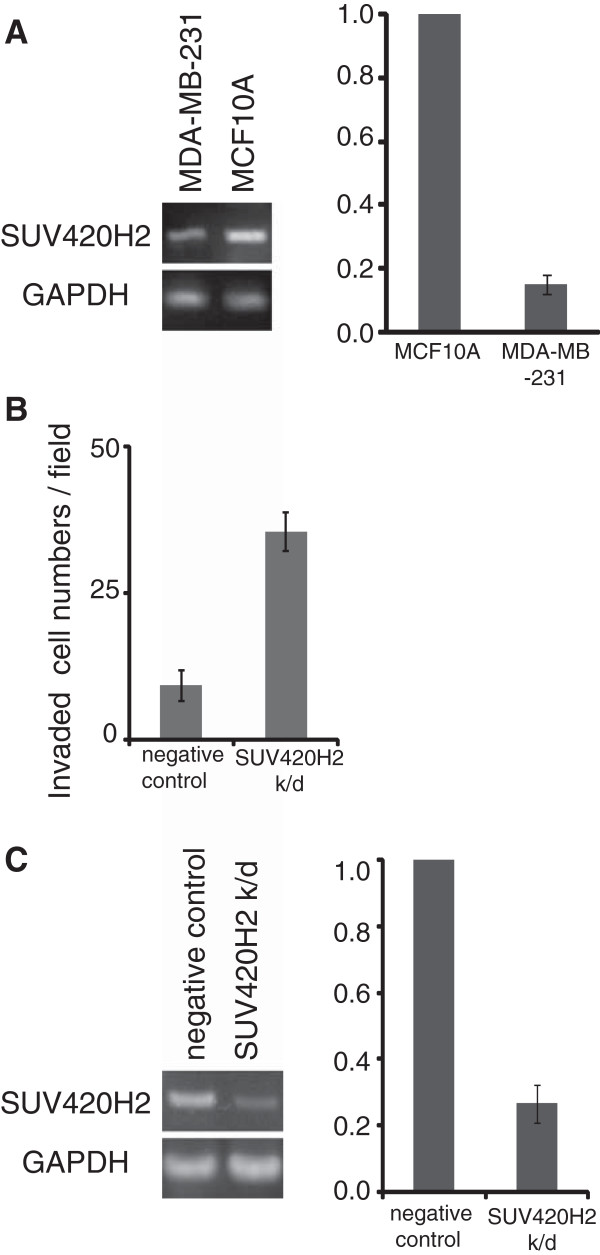
**Knockdown of SUV420H2-activated cell invasion in normal mammary epithelial cells, MCF10A. (A) ***SUV420H2* and *GAPDH* mRNA levels in MCF10A were analyzed by PCR and agarose gel (left) and real-time PCR (right). **(B)** Invasive activities of SUV420H2-knockdown cells. Results are presented as means ± standard deviation (SD). **(C)** siRNA against *SUV420H2* was transfected into MCF10A, and the expression levels of *SUV420H2* and *GAPDH* mRNA were examined by PCR and agarose gel (left) and real-time PCR (right).

Cell invasion is a critical step in tumor metastasis. Our results suggest a negative correlation between invasiveness and the level of H4K20me3 expression, as evidenced by the reduction of H4K20me3 level in invasive breast tumor (Table 1), a positive correlation between loss of H4K20me3 and reduction in disease-free survival, which was associated with metastasis (Figure 2B and Table 2), and lower expression of SUV420H2 in more aggressive MDA-MB-231 and BT-474 breast cancer cells (Figure 3A). Therefore, we investigated the functional relationship between global loss of H4K20me3 and cancer cell invasion by monitoring the expression of SUV420H1 and SUV420H2, which regulate the global level of H4K20me3 [24]. Overexpression of SUV420H1 and SUV420H2 caused clear reduction in the invasive activity of both cell lines (Figure 3D). It is to be noted that BT-474 and MDA-MB-231 cells are HER2-positive and -negative, respectively [31].To confirm the effect of SUV420H2 on cell invasion, we used MCF10A, an immortalized but nontumorigenic mammary epithelial cell line widely used as a normal control for breast cancer cells. These cells express higher levels of SUV420H2 (Figure 4A). Knockdown of SUV420H2 in MCF10A upregulated cell invasion (Figure 4B and C). Taken together, these results suggest that the H4K20me3 level may play a role in determining the invasive activity of cancer cells.

## Discussion

This study reveals three main findings. First, there is a striking reduction of H4K20me3 level in breast cancer tissue. Second, loss of H4K20me3 is a marker of poor prognosis in breast cancer. Moreover, our data also show that the level of H4K20me3 is involved in the regulation of cell invasion. This is the first evidence indicating that the H4K20me3 level may be associated with cancer-cell invasiveness.

### H4K20me3 level is reduced in breast cancer

Loss of H4K20me3 is a hallmark of various cancers, and predicts poor prognosis of several types of cancer other than breast cancer [10,13-15]. Here we showed that, as in other tumor types, most breast cancer cells (63.4%, n = 93) contained less trimethylated H4K20 than noncancerous mammary epithelial cells (Figure 1 and Table 1) or benign tumor cells (Figure S1 in Additional file 2). Some data contradicting these findings has been reported, including a study showing that most cases (69.8%) of invasive breast tumors exhibit intense H4K20me3 staining [18]. This discrepancy may be due to the different image-analysis method used by those authors: they performed semiquantitative assessment of both the intensity of staining and the percentage of positive cells using tissue microarrays and digital image analysis [18], whereas we performed manual scoring that depended only on the cancerous region in each specimen. Because malignant cells with low levels of H4K20me3 staining were often surrounded by noncancerous regions with high levels of H4K20me3 (Figure 1C), these specimens would score higher if the noncancerous regions were not excluded manually. Alternatively, the discrepancy might be related to differences in the genetic backgrounds of the specimens [32]. In any case, our data also demonstrate loss of H4K20me3 in the breast cancer tissue associates with clinicopathological status (Table 1) and poor prognosis (Table 2, Figure 2, discussed below). These results are supportive of the finding that H4K20me3 is lost in breast cancer.

### Loss of H4K20me3 could be a marker of poor prognosis in breast cancer

Our results demonstrate that H4K20me3 levels are associated with the patient outcomes in breast cancer. In particular, low-staining cases exhibited a reduction in disease-free survival rate relative to the high-staining cases (Figure 2), and multivariate analysis also revealed an independent statistically significant association between H4K20me3 and disease-free survival (Table 2). These results collectively indicate that loss of H4K20me3 is a potential independent marker of poor prognosis in patients with breast cancer.

### Association between H4K20me3 level, hormone-receptor status, and disease-free survival

We showed a distinct relationship between H4K20me3 level and disease-free survival (Figure 2 and Table 2). In addition, we also demonstrated a positive relationship between H4K20me3 level and status of hormone receptors such as ER and PgR, but not HER2 (Table 1). Breast cancer can be divided into two intrinsic subtypes in regard to hormone-receptor expression: luminal and non-luminal. The luminal subtype includes ER- and/or PgR-positive cases, and non-luminal subtypes include ER- and PgR-negative cases, regardless of HER2 expression. Luminal subtypes are associated with less aggressive metastatic disease and longer disease-free survival than non-luminal subtypes [4]. The luminal subtype can be divided into two groups, luminal A (HER2-negative) and luminal B (HER2-positive); of the two, luminal A is less aggressive [5]. H4K20me3 level associates with intrinsic subtypes including luminal A and B (*P* = 0.002) more strongly than either ER (*P* = 0.027) or PgR (*P* = 0.019) alone, and independently associates with disease-free survival, but not with luminal A and luminal B distribution (Table S3 in Additional file 4). At this point, however, the causal relationships between H4K20me3, intrinsic subtypes, and disease-free survival remain unclear at the molecular level.

These results raise the question of whether H4K20me3 regulates ER/PgR expression or vice versa. If H4K20me3 acted upstream of ER and PgR expression, ER and PgR expression levels would exhibit the same associations, but this is not the case. Thus, we can envisage a scenario in which hormone-receptor signaling (reflected in either ER or PgR status) could positively regulate H4K20me3.

### A possibility: loss of H4K20me3 could be involved in regulation of cell invasion independently of the HER2 signaling pathway

The pathological functions of H4K20me3 reduction in cancer cells have been explained by proposing that loss of H4K20me3 induces genome instability and is related to DNA hypomethylation [17,33,34]. In addition to these known functions of H4K20me3, here we demonstrated a strong negative correlation between H4K20me3 and invasiveness: ectopic expression of SUV420H1 and SUV420H2 increased H4K20me3 level and concomitantly repressed cancer-cell invasion. This is the first report to demonstrate a pathological function of loss of H4K20me3 in cell invasion, which could promote metastasis *in vivo*. The results of previous studies support this idea: HBL-100 and MCF-7 cells, which endogenously express higher levels of SUV420H2 (Figure 3A), are less invasive than BT-474 and MDA-MB-231 cells [35,36], which express SUV420H2 at lower levels.

H4K20me3 is a repressive mark related to heterochromatin formation [25,26]. Another heterochromatin-associated histone modification, H3K9me3 has been implicated in cell migration [19,37-39]. Several cancers, such as colon cancer, exhibit global upregulation of H3K9 methylation during cancer progression. Recently we observed that upregulation of H3K9me3 activates cell migration [19]; in this study, however, we did not identify any association between H3K9me3 status in breast cancer tissues and clinicopathological status, or between H3K9me3 status and patient survival (Table 1 and Figure 2). Therefore, we concluded that H4K20me3-associated invasiveness is independent of cell migration induced by H3K9me3.

HER2 expression and activity are well known to confer invasive and metastatic ability on breast cancer cells [40-42]. For the following three reasons, however, we hypothesize that H4K20me3-associated invasiveness is independent of the HER2 signaling pathway. First, loss of H4K20me3 is not associated with HER2 expression. Second, loss of H4K20me3 is correlated with non-luminal subtype, in which HER2 expression is irrelevant (Table 1). Third, overexpression of SUV420H1 and SUV420H2 suppressed cell invasion in both HER2-positive (BT-474) and -negative (MDA-MB-231) cells. Together, these results indicate that H4K20me3-associated invasiveness is independent of the HER2 signaling pathway, although the underlying molecular mechanism remains unknown.

Several lines of evidence support a relationship between histone modifications and cancer invasion: overexpression of EZH2, which catalyzes histone H3 lysine 27 trimethylation (H3K27me3), is associated with prostate- and breast-cancer aggressiveness [43-45]; and G9a, which catalyzes histone H3 lysine 9 dimethylation (H3K9me2), promotes lung-cancer invasion [46]. These enzymes promote cancer-cell invasion by repressing cell-adhesion molecules such as E-cadherin and Ep-CAM. Thus, one plausible explanation for the association between global loss of H4K20me3 and cell invasion is that H4K20me3 represses some key genes that are required for the suppression of cell invasion. Although H4K20me3 is associated with repressed chromatin at the centromere and telomere, recent studies have demonstrated the existence of H4K20me3-mediated transcriptional repression [47-49].

### How does the H4K20me3 level decrease in breast tumors?

There are several possible explanations for the decreased H4K20me3 level in breast cancers. A recent chemogenetic analysis revealed that the human genome encodes 96 protein methyltransferases, which can be classified into two families [50]. According to cancer genome databases, at least 20 of these enzymes are misregulated or genetically altered in hematological or solid malignancies [51]. Therefore, H4K20me3 might be lost because the expression of the H4K20-specific histone methyltransferases, SUV420H1 and SUV420H2, might be decreased, as previously reported for cancers of other tissues [15,17]. Indeed, the contents of the public database MENT (see above) revealed that SUV420H2 expression is reduced in breast cancer tissue. Alternatively, the activity of SUV420H1 and/or SUV420H2 might be decreased. It is also possible that a histone demethylase specific for H4K20me3, as yet unidentified, might contribute to H4K20me3 reduction. Future studies should investigate the causes of H4K20me3 reduction in breast cancer tissues, as these findings would contribute to the development of new treatments for breast cancer that suppress invasion by preventing of the loss of H4K20me3.

Epigenetic alterations, including DNA methylation and histone modifications, are thought to play important roles in carcinogenesis and cancer progression. Therefore, many drugs targeted at epigenetic pathways, such as DNA methyltransferase and histone deacetylase inhibitors, have been developed, and some have been approved as cancer therapies [52]. Our results suggest that aberrant H4K20me3 levels affect cancer-cell invasiveness, and are therefore associated with breast cancer progression. The level of H4K20me3 is potentially useful as a prognostic marker in novel strategies that target metastasis.

## Conclusions

In this study, we demonstrated that the loss of H4K20me3 is a novel candidate for an independent prognostic marker for use in breast cancer patients. Moreover, the level of H4K20me3 influences cancer cell invasiveness *in vitro*; therefore, this protein may be involved in regulation of breast cancer progression *in vivo*. Our results also indicate that the H4K20me3-associated invasiveness is independent of the HER2-signaling pathway.

## Abbreviations

BSA: bovine serum albumin; CK: cytokeratin; DCIS: ductal carcinoma *in situ*; DMEM: Dulbecco’s modified Eagle’s medium; EGFR: epidermal growth factor receptor; ELISA: enzyme-linked immunosorbent assay; ER: estrogen receptor; FBS: fetal bovine serum; HER2: human epidermal growth factor receptor; IDC: invasive ductal carcinoma; IgG: immunoglobulin G; mAbs: monoclonal antibodies; PgR: progesterone receptor; siRNA: small interfering RNA; SUV: suppressor of variegation EZH2 (enhancer of zeste homolog 2).

## Competing interests

The authors declare that they have no competing interests.

## Authors’ contributions

YY, AM, YN, MHi, and NM designed the experiments. KY and MT performed immunohistochemical experiments and analysis of patient data. YY, AM, YS, EO, MHa, MHi, and HK performed immunochemical staining and all *in vitro* experiments. MH wrote the manuscript. All authors read and approved the manuscript.

## Supplementary Material

Additional file 1: Table S1 Clinical subtype of patients. The detailed clinical subtypes of the patients used in this study. Click here for file

Additional file 2: Figure S1 H4K20me3 staining in benign tumor tissue. Specimens of benign tumor tissue were stained using anti-H4K20me3 (left) and HE staining (right). Click here for file

Additional file 3: Table S2 H4K20me3 staining score associates with subtype. H4K20me3 staining score was classified by the hormone receptor expression. Click here for file

Additional file 4: Table S3 H4K20me3 staining and Luminal A/Luminal B distribution. H4K20me3 staining score was classified by the Luminal A and Luminal B. H4K20me3 staining score did not associate with Luminal A/Luminal B distribution. Click here for file

Additional file 5: Figure S2 Kaplan-Meier analysis of estrogen receptor expression in breast cancer patients. The patient overall survival time and disease-free survival rate were compared between the estrogen receptor expression low- and high-staining groups by Kaplan-Meier analysis. Click here for file
