# Supplementary material for: Loss of histone H4K20 trimethylation predicts poor prognosis in breast cancer and is associated with invasive activity
Source: Breast Cancer Res. 2014 Jun 22;16(3):R66. doi: 10.1186/bcr3681 (PMC4229880; doi:10.1186/bcr3681)
Supplement: Additional file 1: Table S1 — Clinical subtype of patients. The detailed clinical subtypes of the patients used in this study. [file bcr3681-S1.doc]

Supplemental Table 1.

Clinical subtype of patients.

|  | Total patients | Used for H3K9me3 analysis | Used for H4K20me3 analysis |
| --- | --- | --- | --- |
| Luminal A | 57 | 36 | 46 |
| Luminal B | 11 | 8 | 10 |
| HER2 type | 16 | 11 | 13 |
| Triple negative | 23 | 14 | 22 |
| sunknown | 2 | 2 | 2 |

- Three benign and 109 tumor tissues from 112 patients were obtained.
- Total 71 and 93 tumor samples were used for anti-H3K9me3 and anti-H4K20me3 analysis, respectively in table1 and table 2.
