# Supplementary figures and images for: Loss of histone H4K20 trimethylation predicts poor prognosis in breast cancer and is associated with invasive activity
Source: Breast Cancer Res. 2014 Jun 22;16(3):R66. doi: 10.1186/bcr3681 (PMC4229880; doi:10.1186/bcr3681)

Figure S1 Yokoyama et al.,  
H4K20me3 staining in benign tumor tissue

H4K20me3

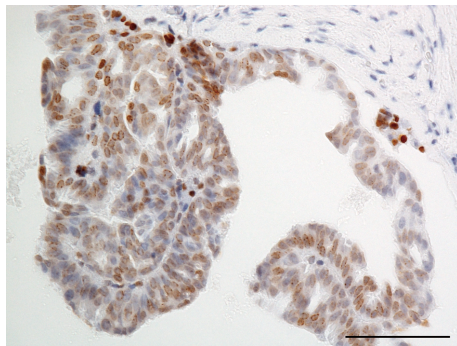

HE staining

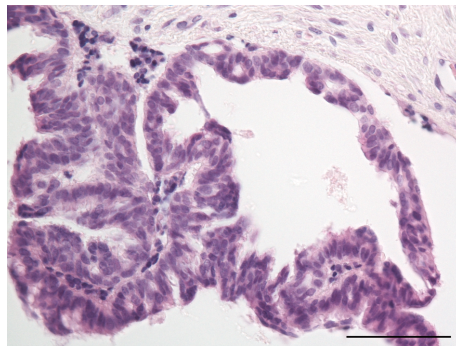

Supplement: Additional file 2: Figure S1 — H4K20me3 staining in benign tumor tissue. Specimens of benign tumor tissue were stained using anti-H4K20me3 (left) and HE staining (right). [file bcr3681-S2.pdf]

Yokoyama et al. Figure S2  
Kaplan-Meier analysis of ER expression in breast-cancer patients

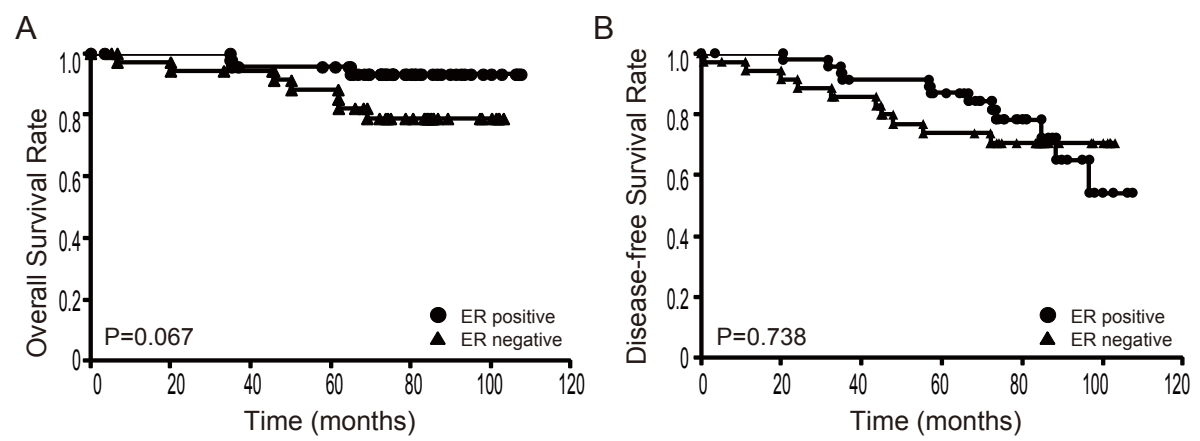

Supplement: Additional file 5: Figure S2 — Kaplan-Meier analysis of estrogen receptor expression in breast cancer patients. The patient overall survival time and disease-free survival rate were compared between the estrogen receptor expression low- and high-staining groups by Kaplan-Meier analysis. [file bcr3681-S5.pdf]
