# Supplementary material for: Loss of histone H4K20 trimethylation predicts poor prognosis in breast cancer and is associated with invasive activity
Source: Breast Cancer Res. 2014 Jun 22;16(3):R66. doi: 10.1186/bcr3681 (PMC4229880; doi:10.1186/bcr3681)
Supplement: Additional file 3: Table S2 — H4K20me3 staining score associates with subtype. H4K20me3 staining score was classified by the hormone receptor expression. [file bcr3681-S3.doc]

Supplemental Table 2.

H4K20me3 staining score associates with subtype

H4K20me3

|  | N | 0 | 1+ | 2+ | P*=0.004 |
| --- | --- | --- | --- | --- | --- |
|  |  |  |  |  |  |
| Luminal A | 46 | 12 | 10 | 24 |  |
| Luminal B | 10 | 2 | 4 | 4 |  |
| HER2 | 13 | 5 | 6 | 2 |  |
| Triple negative | 22 | 15 | 3 | 4 |  |

*Chi-squared test
