# Supplementary material for: Loss of histone H4K20 trimethylation predicts poor prognosis in breast cancer and is associated with invasive activity
Source: Breast Cancer Res. 2014 Jun 22;16(3):R66. doi: 10.1186/bcr3681 (PMC4229880; doi:10.1186/bcr3681)
Supplement: Additional file 4: Table S3 — H4K20me3 staining and Luminal A/Luminal B distribution. H4K20me3 staining score was classified by the Luminal A and Luminal B. H4K20me3 staining score did not associate with Luminal A/Luminal B distribution. [file bcr3681-S4.docx]

Supplemental Table 3.

H4K20me3 staining and Luminal A / Luminal B distribution

H4K20me3

|  | N | 0 | 1+ | 2+ | P* |
| --- | --- | --- | --- | --- | --- |
|  | 56 |  |  |  | 0.4817 |
| Luminal A |  | 12 | 10 | 24 |  |
| Luminal B |  | 2 | 4 | 4 |  |

Total number of luminal A and luminal B is 56.

*Chi-squared test

LuminalA, ER positive and/or PgR positive, HER2 negative

LuminalB, ER positive and/or PgR positive, HER2 positive

Though H4K20me3 staining level correlated with PgR and ER expression (Table 1), it did not correlate with HER2 expression within luminal, ER and/or PgR positive patients. It is consistent with that H4K20me3 staining level did not correlates HER2 expression (Table 1).
